# Preparation and Physicochemical Properties of High-Temperature-Resistant Polymer Gel Resin Composite Plugging Material

**DOI:** 10.3390/gels11050310

**Published:** 2025-04-22

**Authors:** Tao Wang, Weian Huang, Jinzhi Zhu, Chengli Li, Guochuan Qin, Haiying Lu

**Affiliations:** 1 School of Petroleum Engineering, China University of Petroleum (East China), Qingdao 266580, China; wangtao953@126.com; 2 Tarim Oilfield Branch, Korla 841000, China

**Keywords:** drilling fluid, resin gel plugging system, urea-formaldehyde resin, physicochemical properties, pressure plugging capacity

## Abstract

Lost circulation has become one of the important problems restricting the speed and efficiency of oil and gas drilling and production. To address severe drilling fluid losses in high-temperature fractured formations during deep/ultra-deep well drilling, this study developed a high-temperature and high-strength gelled resin gel plugging system through optimized resin matrix selection, latent curing agent, flow regulator, filling material, etc. Comparative analysis of five thermosetting resins revealed urea-formaldehyde resin as the optimal matrix, demonstrating complete curing at 100–140 °C with a compressive strength of 9.3 MPa. An organosilicon crosslsinker-enabled water-soluble urea-formaldehyde resin achieved controlled solubility and flow–cure balance under elevated temperatures. Orthogonal experiments identified that a 10% latent curing agent increased compressive strength to 6.26 MPa while precisely regulating curing time to 2–2.5 h. Incorporating 0.5% rheological modifier imparted shear-thinning and static-thickening behaviors, synergizing pumpability with formation retention. The optimal formula (25% urea-formaldehyde resin, 10% latent curing agent, 10% high-fluid-loss filler, 0.5% rheological modifier) exhibited superior thermal stability (initial decomposition temperature 241 °C) and mechanical integrity (bearing pressure 13.95 MPa in 7 mm wedge-shaped fractures at 140 °C). Microstructural characterization confirmed interlocking crystalline layers through ether-bond crosslinking, providing critical insights for high-temperature wellbore stabilization.

## 1. Introduction

Lost circulation control, which involves the selection of appropriate materials and methods to seal loss zones and mitigate drilling fluid loss, plays a critical role in drilling operations [1]. Diverse lost circulation technologies, such as pill-based and bridging techniques [2,3,4,5], are employed based on specific formation conditions. In deep and ultra-deep fractured reservoirs, drilling fluid loss is highly prevalent, and the integrity of bridging materials is often compromised under high temperature, pressure, and stress, resulting in suboptimal sealing performance [6]. Compared to bridging methods, pill-based techniques are more effective in forming impermeable barriers, thereby enhancing the success rate of initial treatments. Crucially, the selection of appropriate lost circulation materials is fundamental to the success of these methods. Fluid loss plays a critical role in drilling operations because it directly affects the efficiency and safety of reservoir development and has significant economic and environmental consequences [7].

Coagulable lost circulation materials, characterized by their thixotropic and rapid-curing properties, are designed to solidify efficiently within loss zones [8]. Typically comprising curing agents, suspension stabilizers, and retarders, these materials exhibit strong thixotropic behavior and high flow resistance in loss zones, enabling rapid solidification into high-strength masses that bond with the formation to seal fluid loss pathways. Coagulable materials are widely sourced, cost-effective, and easy to prepare, offering robust post-curing mechanical strength.

Existing coagulable lost circulation materials include Sentinel CemTM, developed by Halliburton [9], MAGNE-SET by Baker Hughes [1], China’s high-strength high-temperature chemical binder HDL-4 [10], downhole crosslinking solidification agents [11], and composite materials combining chemical solidification and crosslinking film-forming technologies [12]. Compared to conventional bridging materials, coagulable systems exhibit superior pressure-bearing capacity and reliable curing properties, ensuring the integrity of sealing layers under drilling fluid hydrostatic pressure during circulation, thereby significantly reducing the frequency of remedial treatments [13]. The synergistic use of coagulable materials with inert additives enhances treatment success rates [14]. Inert materials serve as bridging and filling agents, achieving a “sealing gate” effect, while coagulable materials effectively retain within loss zones, mitigating fluid loss and rapidly solidifying into high-strength barriers, thus improving sealing performance [15,16,17]. When combined with crosslinkable polymers, coagulable materials form more robust three-dimensional network structures, optimizing the sealing effect [18,19]. However, current coagulable materials in the drilling fluid industry face limitations such as short curing times, elevated operational risks, susceptibility to dilution by formation water, and inadequate thermal stability, which can lead to downhole complications such as stuck pipes [11,20,21]. These shortcomings significantly restrict the application of coagulable materials in addressing lost circulation challenges in deep and ultra-deep drilling environments [22,23,24,25].

This study focuses on the development of a high-temperature-resistant gelled resin gel plugging system, with a thermally stable resin material as the core component. By incorporating rheological modifiers, the fluidity and retention of the lost circulation agent prior to solidification are optimized. Additionally, key components such as filling materials and curing agents are introduced to enhance the structural strength and thermal resistance of the system. The method aims to construct a high-strength resin gel plugging system with a temperature resistance of 140 °C, which can cope with severe drilling fluid loss challenges in a harsh downhole environment.

## 2. Results and Discussion

### 2.1. Development of a High-Temperature-Resistant High-Strength Gelled Resin Gel Plugging System

#### 2.1.1. Optimization of High-Temperature-Resistant Resin Substrates

In this study, the curing performance characteristics of five typical thermosetting resins (urea-formaldehyde resin, epoxy resin, phenolic resin, polyvinyl alcohol resin, and melamine resin) were systematically investigated. Controlled experiments were conducted at three temperature gradients (60 °C, 80 °C, and 100 °C) by adding an appropriate amount of curing agent to observe their curing behavior. The results (Table 1) indicate that none of the tested resins exhibited curing behavior at an ambient temperature of 40 °C. As the temperature increased, urea-formaldehyde resin, phenolic resin, polyvinyl alcohol resin, and epoxy resin all demonstrated varying degrees of curing tendency, while melamine resin failed to achieve effective curing within the 40–100 °C temperature range. Notably, under 100 °C curing conditions, urea-formaldehyde resin and phenolic resin exhibited excellent curing strength and mechanical properties. In contrast, the curing degree of polyvinyl alcohol resin and epoxy resin was significantly reduced, and their mechanical performance failed to reach the desired levels.

**Figure 1:**
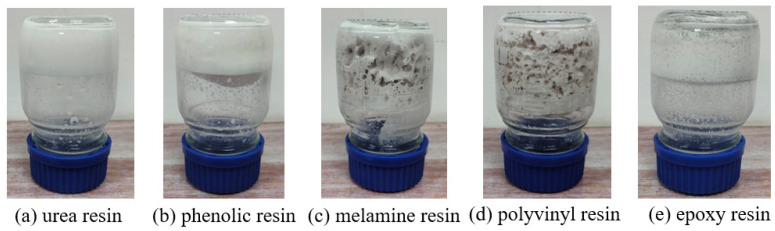
Results of static curing of five resins at 60 °C.

The curing profiles of five thermosetting resins were systematically evaluated under isothermal conditions (60 °C). As illustrated in Figure 1, the urea-formaldehyde resin and phenolic resin showed the best curing properties, and the cured products had a complete network structure and good mechanical properties. These variations strongly correlate with molecular architecture and temperature-dependent crosslinking efficiency. Conversely, epoxy, polyvinyl alcohol, and melamine resins showed incomplete crosslinking under identical thermal activation, indicating restricted network formation. The results demonstrate temperature-mediated control over resin curing dynamics while establishing selection criteria for thermosetting systems in moderate-temperature applications.

Further optimization was conducted by selecting urea-formaldehyde resin, epoxy resin, and phenolic resin as the primary candidates. Each resin matrix was added to water and uniformly dispersed by stirring at 600 rpm for 5–15 min. Subsequently, thiourea, lithium-based bentonite, curing agent, crosslinking agent, nano-silica, walnut shell, and quartz sand were incorporated. The choice of curing agent was tailored to the specific resin: triethanolamine for epoxy resin, sodium hydroxide for phenolic resin, and a latent curing agent composed of sulfonic acid and ammonium compounds for urea-formaldehyde resin. The mixture was further stirred for 5–15 min until uniformly dispersed, then poured into cylindrical high-temperature-resistant molds. The molds were placed in a variable-frequency high-temperature roller heating furnace and subjected to rolling heating at 100–140 °C for a specified duration. After removal, the curing status was observed.

From the curing point of view, under the same experimental conditions (100–140 °C), epoxy resin and phenolic resin failed to meet the molding requirements, and the strength requirements required for pressure sealing, while urea-formaldehyde resin has the best performance among the three thermosetting resins, with good molding and certain strength, combined with its good settling stability. Finally, the urea-formaldehyde resin was selected as the resin matrix for this experiment.

#### 2.1.2. Preparation of Water-Soluble Resin

In order to meet the application requirements of an underground in situ cross-linked plugging agent, a water-soluble resin with controllable curing properties was prepared by using urea-formaldehyde resin as the target resin matrix through innovative synthesis technology. Compared with traditional resins, which have uncontrollable curing times and high costs at high temperatures, water-soluble resins not only have good water solubility but also have better curing process adaptability.

The synthesis process of the water-soluble resin comprises three critical steps:

1. Preparation of Prepolymer. Urea, formaldehyde, and additives such as resorcinol and furfural are mixed at specific molar ratios, followed by pH adjustment to initiate the condensation polymerization reaction;
2. Hydrophilic Modification. Utilizing an internal emulsification method, hydrophilic groups or chain extenders containing hydrophilic groups are introduced into the prepolymer to achieve chemical modification;
3. Crosslinking and Curing. Multifunctional organosilicon compounds are selected as crosslinking agents. Their unique structure enables the formation of covalent bonds with both the active groups of the resin (hydroxymethyl groups, amide bonds, etc.) and the surface groups of inorganic materials, thereby constructing a crosslinked network.

**Figure 2:**
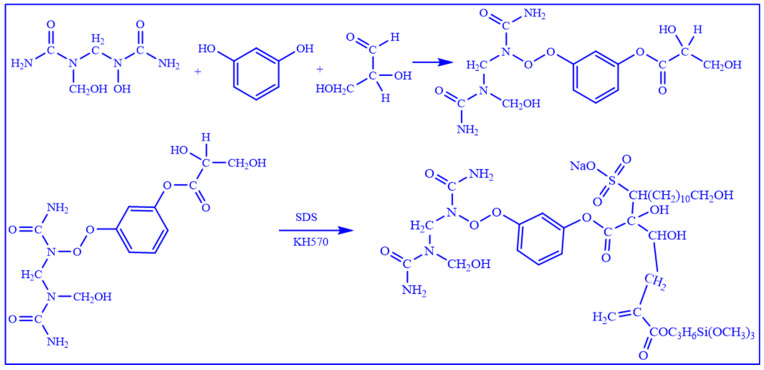
The chemical reaction formula of the preparation process of water-soluble resin.

The reaction process and microstructure of the water-soluble resin preparation are illustrated in Figure 2. Through precise control of the prepolymerization reaction, the introduction of hydrophilic groups, and the use of multifunctional crosslinking agents, a water-soluble resin gel system with both excellent fluidity and controllable curing properties was successfully synthesized. This achievement provides a reliable material foundation for the practical application of in situ leakage control agents in subsurface environments.

An orthogonal experimental design was employed to systematically assess the effects of chemical component ratios on the synthesis of water-soluble resins. Through rigorous multi-factor analysis, the optimal formulation parameters (summarized in Table 2) were determined as follows: resorcinol at 0.5 wt%, furfural at 1.0 wt%, sodium dodecyl sulfate at 0.6 wt%, and organosilicon crosslinking agent at 0.3 wt%. This optimized combination maximizes intermolecular interactions between constituents, significantly enhancing the resin’s structural uniformity, solution stability, and adhesion properties. The systematic screening approach further reveals dose-dependent relationships between additives and functional performance, providing quantitative guidance for scalable production while maintaining targeted application specifications.

#### 2.1.3. Optimization of the Type and Dosage of Curing Agent

To prevent premature curing of the leakage control system during the pumping process, the curing agent was optimized to extend the curing time of the resin gel plugging system, thereby ensuring operational safety. Three types of curing agents—ammonium-based curing agents, latent curing agents, and mixed ammonium-based curing agents—were selected and compared for their effectiveness in curing the resin aqueous solution. The curing temperature was maintained at 130 °C, and the curing time was set at 180 min for all tests.

**Figure 3:**
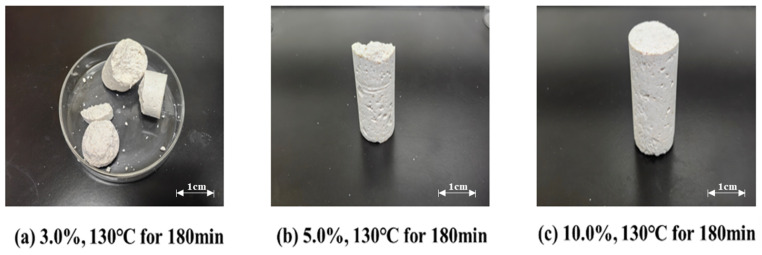
Curing conditions of different concentrations of ammonium chloride curing agent.

The curing performance of resin-mortar composites under thermal compression (130 °C, 180 min) was systematically analyzed as a function of ammonium-based hardener concentration (3–10 wt%). As depicted in Figure 3, lower hardener dosage (3 wt%) produced structurally heterogeneous consolidations with compromised mechanical strength, indicative of insufficient crosslinking density. Elevated concentrations (5–10 wt%) facilitated uniform network formation, yielding cohesive composites with enhanced structural integrity and load-bearing capacity. This concentration-dependent behavior aligns with thermally activated crosslinking kinetics, where adequate hardener availability promotes complete covalent bonding between polymeric chains. The findings establish critical thresholds for additive optimization in high-temperature resin processing and industrial composite fabrication.

**Figure 4:**
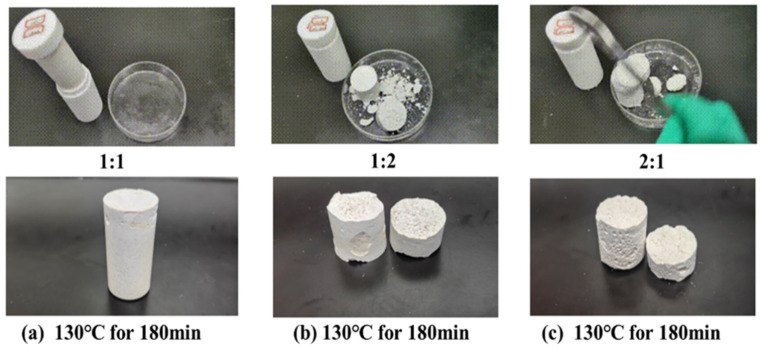
Curing conditions of mixed curing agents with different ratios of ammonium persulfate and diammonium hydrogen phosphate.

The impact of different ratios of mixed curing agents (ammonium persulfate and diammonium hydrogen phosphate) on the curing effectiveness of the resin gel plugging system was studied under the conditions of 130 °C and 180 min of thermal rolling. The ratios of ammonium persulfate to diammonium hydrogen phosphate in the mixed curing agents were set at 1:1, 1:2, and 2:1, respectively. The experimental results, as depicted in Figure 4, reveal that a mixed curing agent ratio of 1:1 resulted in the formation of a complete resin mortar consolidation with high strength. In contrast, ratios of 1:2 and 2:1 produced consolidations that exhibited brittleness.

Ammonium persulfate (APS) served dual roles as catalyst and initiator for the urea-formaldehyde resin curing process, mediated by its unique redox-active coordination chemistry. Thermal activation facilitated APS decomposition into sulfate radicals that abstracted hydrogen atoms from the resin’s hydroxymethyl/methylene moieties, inducing radical chain reactions. These reactive intermediates promoted covalent crosslinking via ether/methylene bridge formation between polymeric chains, driven by radical recombination mechanisms. Concomitantly, APS’s acidic dissociation products lowered the system pH, catalytically accelerating polycondensation through the protonation of hydroxyl groups. The synergistic action of radical-mediated crosslinking and acid-catalyzed polycondensation generated a densely crosslinked, interpenetrated network architecture with enhanced thermomechanical stability. This dual-functional mechanism underscores APS’s efficacy in controlling both reaction kinetics and final network topology during thermosetting resin matrix consolidation. The findings elucidate redox initiator design principles for tailoring covalent adaptable networks in functional polymer composites.

**Figure 5:**
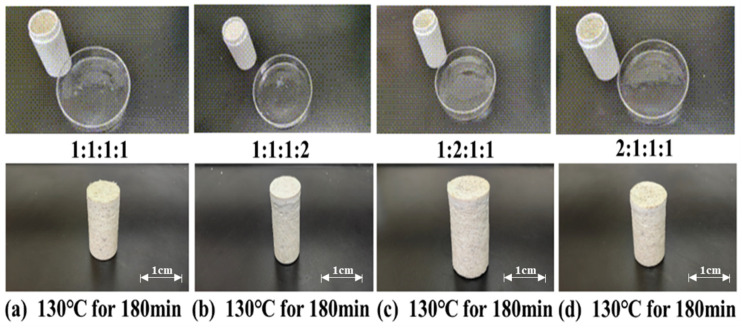
Curing conditions of different proportions of latent curing agents.

The influence of different ratios of latent curing agents on the curing effectiveness of the resin gel plugging system was investigated under the conditions of 130 °C and 180 min of thermal rolling. The latent curing agents were composed of p-toluenesulfonic acid, hexamethylenetetramine, diethanolamine, and ammonium persulfate, with the ratios set at 1:1:1:1, 1:1:1:2, 1:2:1:1, and 2:1:1:1, respectively. The experimental results, as illustrated in Figure 5, demonstrate that the resin gel plugging system achieved the highest strength when the latent curing agent ratio (p-toluenesulfonic acid: hexamethylenetetramine: diethanolamine: ammonium persulfate) was 1:1:1:2. While the resin gel plugging system also solidified at ratios of 1:1:1:1, 1:2:1:1, and 2:1:1:1, the resulting consolidations exhibited lower strength.

The superior mechanical performance at the 1:1:1:2 stoichiometric ratio arises from balanced acid-base dynamics, combining reduced p-toluenesulfonic acid (PTSA) content with elevated ammonium persulfate (APS) levels. This formulation leverages the latent curing behavior of APS, which maintains resin-curing agent metastability at ambient conditions. Upon thermal or photonic stimulation, APS undergoes heterolytic cleavage, activating reactive sulfate radicals that abstract hydrogen atoms from the resin’s hydroxymethyl/methylene functionalities. These radical intermediates drive covalent crosslinking via radical chain-transfer mechanisms, while residual p-toluenesulfonic acid catalyzes methylene bridge formation through Friedel–Crafts alkylation. The synergistic interplay between radical-mediated polymerization and acid-catalyzed polycondensation induces stereochemically directed 3D network assembly, converting linear oligomers into a glassy thermoset matrix. The optimized curing kinetics minimizes stress-induced microcracks, producing homogeneous architectures with superior interfacial adhesion. These structure–property relationships demonstrate rational formulation design for stimuli–responsive thermoset systems requiring delayed activation and high mechanical fidelity.

The compressive strength of the resin varies depending on the materials and processes used. To evaluate the compressive strength, samples of the resin gel plugging system with the highest strength were selected from those formed using three types of curing agents: ammonium chloride curing agent, latent curing agent, and a mixed curing agent of ammonium persulfate and diammonium hydrogen phosphate. The experimental results, as presented in Table 3, indicate that the latent curing agent yielded the best compressive strength, reaching up to 6.26 MPa. Consequently, the latent curing agent was chosen for subsequent experiments due to its superior performance in enhancing the mechanical properties of the resin gel plugging system.

**Figure 6:**
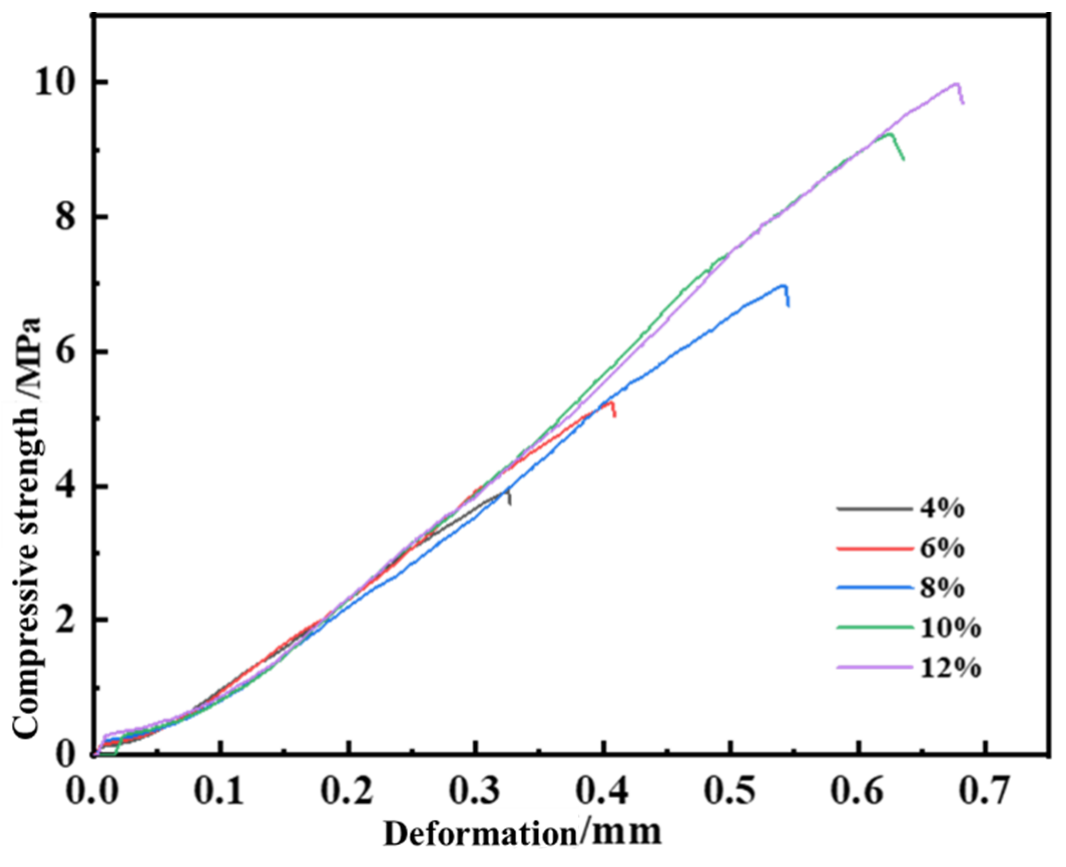
Pressure bearing capacity of leakage plugging slurry with different curing agent dosages.

The curing agent concentration critically influenced the apparent viscosity, bearing capacity, and curing time of the leakage control slurry. By adjusting its dosage, the slurry’s comprehensive performance was optimized. Injections were performed into a steel fracture (7 mm inlet, 5 mm outlet), allowing in situ curing. Post-curing, the bearing capacity was evaluated using a leakage control instrument with incremental pressure application. Compressive strength, bearing strength, and apparent viscosity were selected as key metrics for assessment. A single-factor analysis method was employed to determine the optimal curing agent concentration, as summarized in Table 4. Experimental outcomes are visualized in Figure 6, demonstrating the critical role of precise stoichiometric control in achieving balanced rheological and mechanical properties for fracture-sealing applications.

The compressive strength of the consolidated body increased proportionally with curing agent dosage. A critical enhancement in bearing strength was observed when the dosage rose from 8% to 10%, increasing significantly from 6.3 MPa to 9.2 MPa. Comparative analysis revealed nearly identical compressive strengths at 10% and 12% dosages, with both formulations demonstrating equivalent high-performance mechanical resistance under compressive loads.

**Figure 7:**
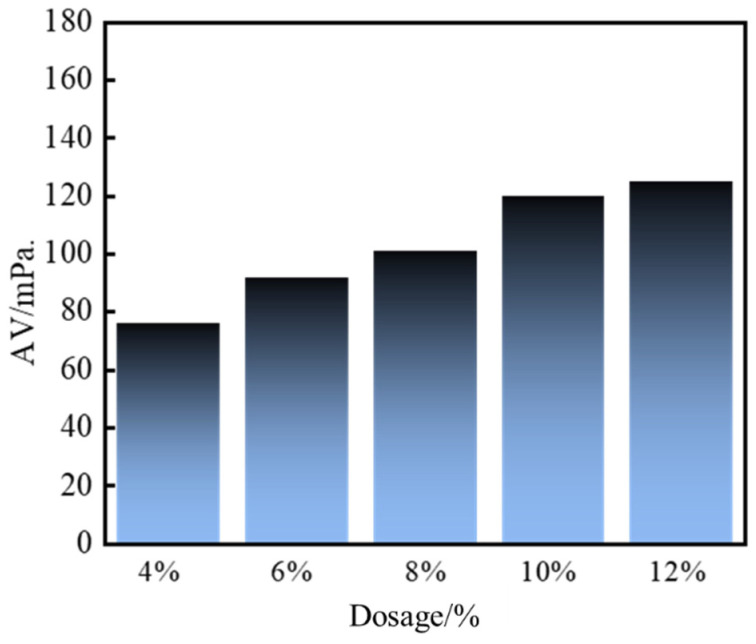
Apparent viscosity of sealing slurry with different curing agent content.

**Figure 8:**
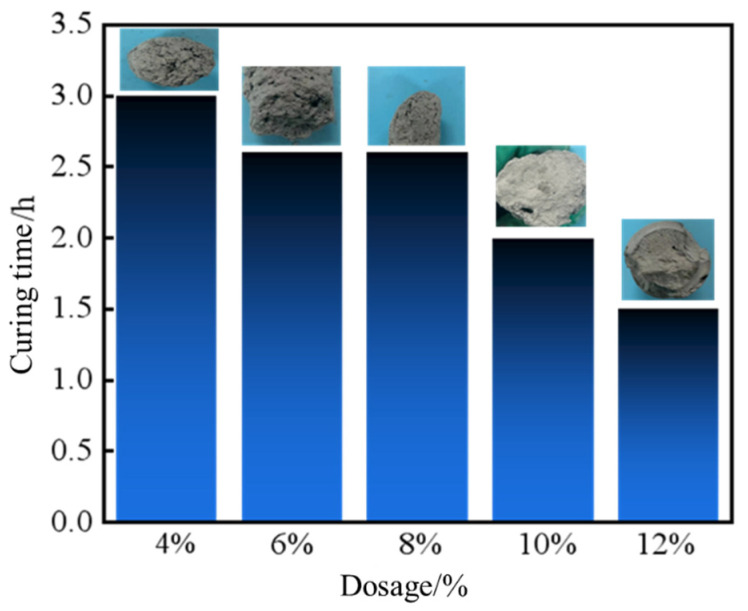
Curing time of sealing slurry with different curing agent contents.

As depicted in Figure 7 and Figure 8, the apparent viscosity of the leakage control slurry increased with curing agent dosage, while the curing time decreased. This relationship correlated with enhanced homogeneity of the consolidated body and improved bearing capacity. At 10% curing agent dosage, a curing time of 2 h and a pressure-bearing sealing strength of 8.0 MPa were achieved. This formulation notably balanced rapid gelation kinetics (to minimize fluid loss during injection) with sufficient strength development for sealing efficacy. Consequently, the optimal curing agent dosage was determined as 10%, striking an equilibrium between workability and structural integrity for fracture remediation.

#### 2.1.4. Construction of Basic Formula for Plugging System

**Figure 9:**
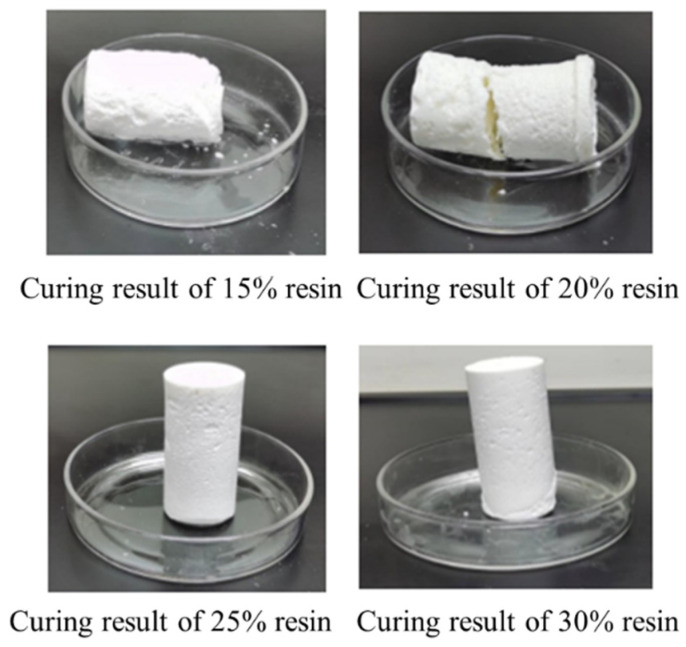
Curing effect of urea-formaldehyde resin with different concentrations.

In this study, an orthogonal experimental approach was employed to optimize the formulation of the resin-based leakage control system, aiming to reduce resin concentration and costs. Under the conditions of a curing temperature of 130 °C and a curing time of 180 min, the effects of urea-formaldehyde resin dosage (10%, 15%, 20%, 25%, 30%) on the curing performance were systematically investigated. The orthogonal experimental design with varying additive dosages is detailed in Table 5, and the experimental results are presented in Figure 9. The results indicate that when the urea-formaldehyde resin dosage was 10% and 15%, the resin underwent initial curing but exhibited insufficient strength, failing to meet practical application requirements. When the dosage was increased to 20% and 25%, the resin successfully cured into intact cylindrical consolidated bodies, demonstrating excellent curing performance and high strength. However, when the dosage was further increased to 30%, the cured samples exhibited fracturing, and the curing strength decreased. Comprehensive analysis revealed that a urea-formaldehyde resin dosage of 25% not only ensured adequate curing strength but also effectively reduced resin usage, achieving the dual objectives of optimizing the formulation and lowering costs.

**Figure 10:**
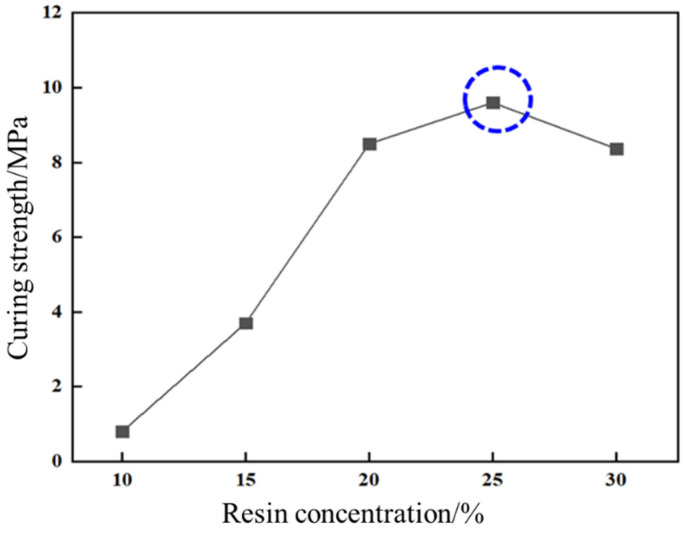
Effect of resin concentration on curing strength.

This study evaluated the impact of resin concentration on curing strength and temperature on curing kinetics. As illustrated in Figure 10, compressive strength exhibited a bell-shaped dependence on urea-formaldehyde resin concentration. At 20%, 25%, and 30% resin concentrations, compressive strengths measured 8.5 MPa, 9.6 MPa (peak performance), and 8.3 MPa, respectively, indicating an optimal concentration threshold at 25%.

**Figure 11:**
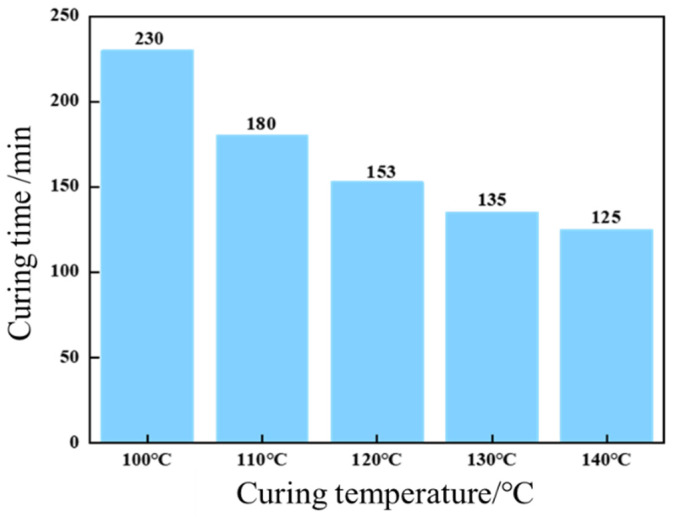
Effect of temperature on curing time.

Concurrently, elevated temperatures significantly accelerated curing kinetics, as demonstrated in Figure 11. Curing time decreased progressively from 230 min at 100 °C to 153 min (120 °C) and 125 min (140 °C), underscoring the thermally activated nature of the curing process. These findings highlight the synergistic interplay between resin formulation and thermal conditions in tailoring cure kinetics and mechanical performance.

The integrated findings establish that a 25% urea-formaldehyde resin concentration, coupled with tailored thermal activation, achieves synergistic optimization of curing performance. This formulation balances rapid curing kinetics (minimizing operational delays) with maximal mechanical strength retention, critical for durable sealing applications. The identified parameters (resin composition and curing temperature) govern the interplay between crosslinking density and network formation dynamics, ensuring robust mechanical integrity under stress. These insights provide a foundational framework for refining resin-based leakage control systems, prioritizing both structural resilience and field-deployable curing efficiency in subsurface engineering applications.

**Figure 12:**
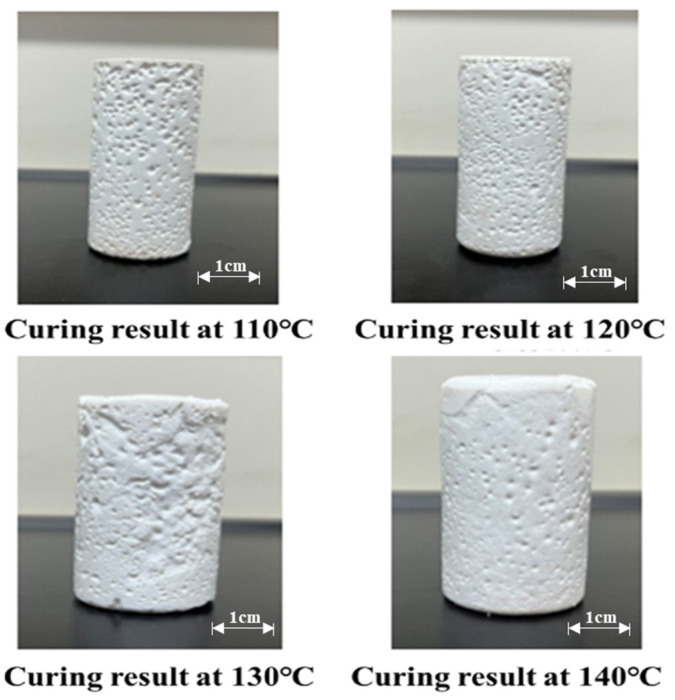
Curing effect of resin plugging material at different temperatures.

Orthogonal experimental analysis optimized the resin-based leakage control formulation to 25% water-soluble resin + 10% curing agent, balancing cost and performance. As shown in Figure 12, modulating resin/curing agent concentrations enabled precise control of curing parameters: temperature (100–140 °C), time (125–230 min), and strength (9–13 MPa), with 25% resin achieving peak strength (9.6 MPa). The formulation optimizes crosslinking kinetics—resin concentration governs polymer network density while curing agent concentration regulates reaction initiation. At 140 °C, curing was completed in 125 min, demonstrating rapid field-applicable polymerization. This synergy of stoichiometric precision and thermal activation provides a low-cost, high-performance solution for engineering applications requiring durable leak prevention.

#### 2.1.5. Dosage Optimization of Flow Regulator

The regulation of the flow behavior of the leakage control agent during its movement is one of the critical technical challenges in ensuring effective leakage control. When the leakage control agent flows through surface piping, it requires low apparent viscosity to ensure pumpability, thereby reducing construction difficulty and energy consumption. Conversely, when flowing within formation fractures, it needs to exhibit a high apparent viscosity to resist the scouring effect of formation water, ensuring the agent can effectively reside and solidify. Therefore, regulating the flow behavior of the leakage control agent holds significant engineering value.

This study utilized a flow behavior regulator developed by the team of Professor Bai Yingrui at China University of Petroleum (East China) [26]. This regulator is a lithium-modified bentonite material with excellent suspension properties, high swelling capacity, and good adsorption performance. Its core advantage lies in the ability to precisely adjust the apparent viscosity of the leakage control agent through simple dosage regulation, thereby meeting the flow behavior requirements under different working conditions. To further investigate the impact of the rheological regulator on the performance of the resin gel plugging system, this study systematically examined the influence of rheological regulator concentration on the system’s shear thixotropy.

**Figure 13:**
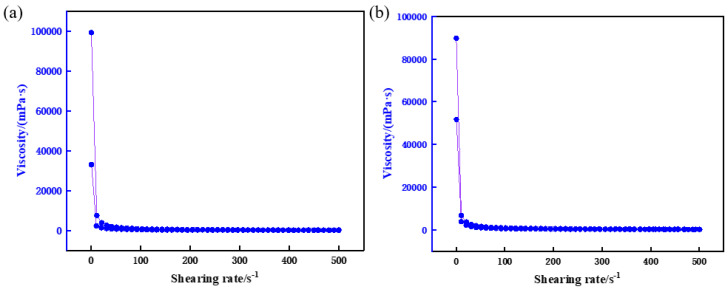
Optimization of rheological regulator concentration. (**a**) 0.5%, (**b**) 1.0%.

The experimental results (Figure 13) reveal that incorporating the rheological regulator imparts distinct “shear-thinning, static-thickening” thixotropic behavior to the resin-gel plugging system. Under high shear rates, the system’s apparent viscosity decreases significantly, enhancing pumpability through surface piping. Conversely, under low shear or static conditions, viscosity rapidly recovers and increases, improving retention within fractures. Critically, the regulator minimally impacts the cured strength of the gel system, ensuring stable leakage control.

The optimal rheological regulator concentration was determined as 0.5%, balancing pumpability (excellent fluidity during injection) with resistance to formation water dilution. This concentration achieves operational efficiency without compromising long-term stability in reservoirs.

#### 2.1.6. Optimal Selection of Filling Materials

In the process of optimizing the leakage control agent formulation, this study systematically screened ten high-water-loss filler materials with different physicochemical properties, including diatomaceous earth, sepiolite, asbestos powder, and others. The screening of these materials was primarily based on their performance in field leakage control operations, focusing on their impact on the water-loss performance, curing strength, and rheological properties of the leakage control agent.

**Figure 14:**
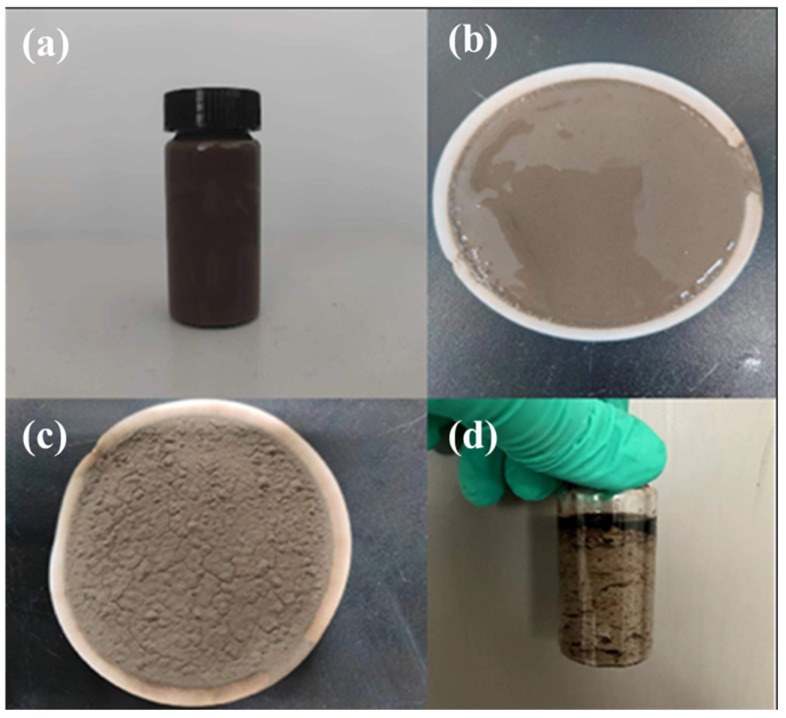
Formation of mud cake and sedimentation curing effect of high-water-loss filling material C. (**a**) Sample before condensation; (**b**,**c**) Mud cake plugging layer; (**d**) The sample after solidification and curing.

High-water-loss filler materials exhibit significant curing and expansion characteristics under dehydration conditions. Introducing these materials into the resin mortar leakage control system enables rapid dehydration and expansion curing under pressure differentials, effectively sealing the target leakage zones. Single-factor optimization experiments revealed that the type of high-water-loss filler material significantly influences the homogeneity and strength of the mudcake. Among them, the leakage control slurry containing high-water-loss filler material C formed a mudcake with significantly improved density, uniform distribution, and excellent toughness, showing no fracturing under bending conditions. The sealing layer exhibited good strength (Figure 14). Therefore, high-water-loss filler material C was selected as the key component of the leakage control slurry system.

**Figure 15:**
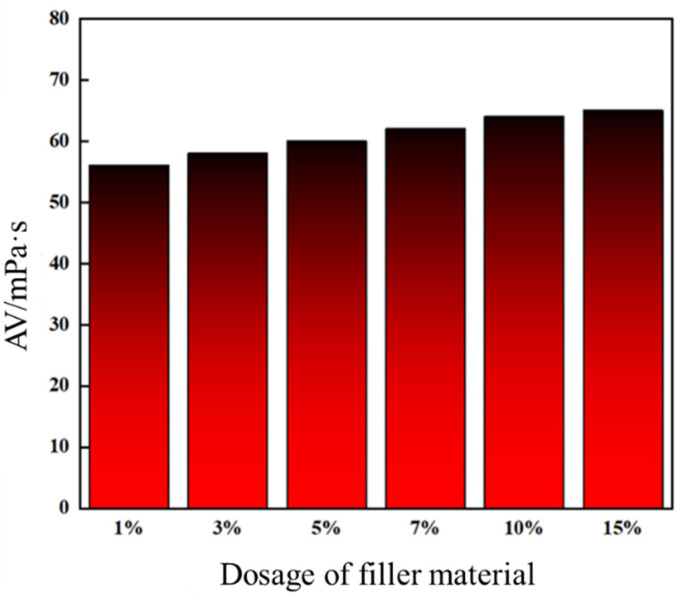
Apparent viscosity of formulations with different high-water-loss filling material additions.

Leakage control slurries formulated with high-water-loss filler materials at varying dosages were evaluated for apparent viscosity, with the experimental matrix outlined in Table 6. As shown in Figure 15, increasing the filler content enhanced consolidated body strength and reduced porosity, improving structural density. Concurrently, the slurries exhibited a progressive rise in apparent viscosity. At 10% and 15% filler dosages, viscosities reached 64 mPa·s and 65 mPa·s, respectively, showing comparable values.

Results demonstrate that filler dosage critically governs slurry rheology (e.g., viscosity modulation) and consolidated performance (e.g., strength–density trade-off). The near-identical viscosities at 10–15% suggest a threshold effect, where further filler addition minimally improves flow behavior while maintaining mechanical enhancement. This highlights the importance of optimizing filler content to balance injectability and plugging efficacy.

**Figure 16:**
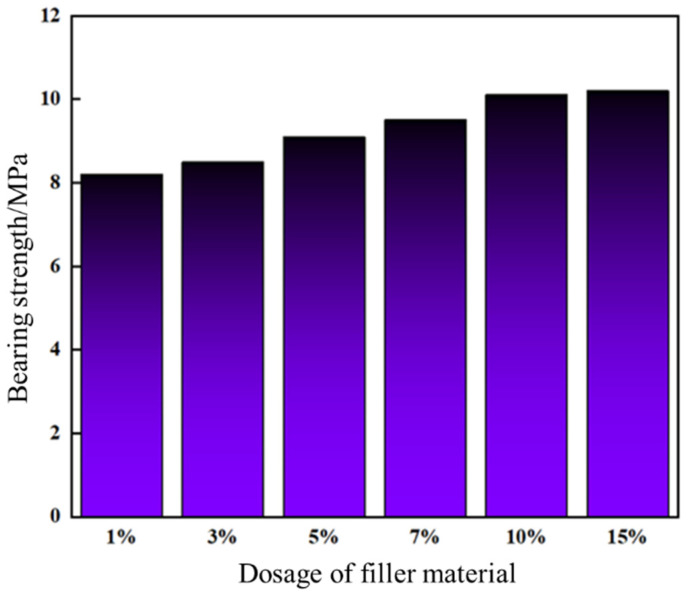
Pressure bearing capacity of plugging slurry with different amounts of high-water-loss filling materials.

The leakage control slurry was injected into a steel-simulated fracture and cured under temperature-controlled conditions. A high-temperature/high-pressure evaluation apparatus performed sequential pressure-loading tests to assess pressure-bearing capacity. As shown in Figure 16, the slurry’s pressure resistance markedly increased with higher high-water-loss filler content. At 10% and 15% filler dosage, the pressure-bearing capacities reached 10.1 MPa and 10.2 MPa, respectively, exhibiting negligible divergence. Post-injection observations confirmed complete sealing at the fracture entrance, with a dense internal structure forming a continuous sealing zone within the fracture.

Considering apparent viscosity (64 mPa·s at 10% vs. 65 mPa·s at 15%), pressure resistance, and cost-effectiveness, the optimal filler dosage was determined as 10%. This dosage balances mechanical robustness (<1% difference in pressure resistance between 10% and 15%) with operational efficiency, achieving both effective fracture sealing and economic feasibility.

#### 2.1.7. Development of a High Temperature-Resistant High-Strength Gelled Resin Gel Plugging System

**Figure 17:**
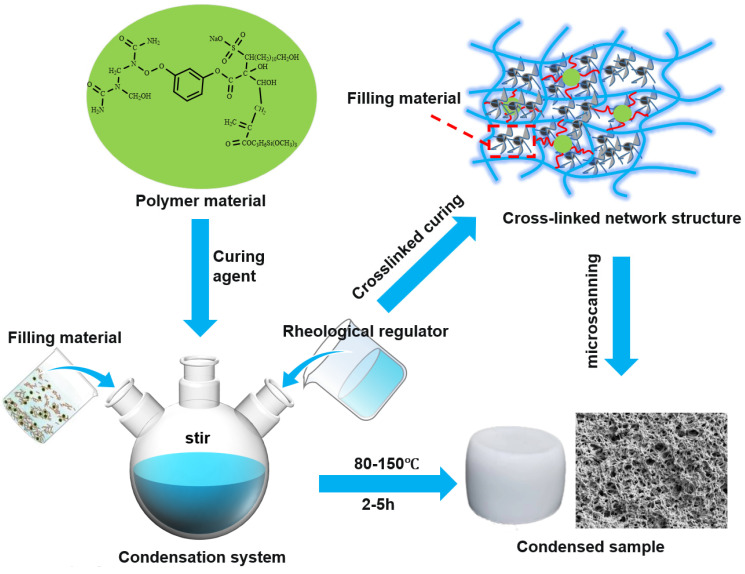
Schematic diagram of construction of resin consolidation and plugging system.

A resin-based leakage control system was engineered with 25% resin, 10% curing agent, 10% filler, 0.5% rheological regulator, and 0.05% dispersant, as detailed in Figure 17. This system achieved a 2.5 h curing time at 140 °C, a density of 1.23 g/cm^3^, an apparent viscosity of 47.5 mPa·s, and a compressive strength of 9.3 MPa. In wedge-shaped fractures (10 mm inlet, 12 mm outlet, 30 cm length), it demonstrated a pressure-bearing capacity of 10.73 MPa, forming a complete sealing zone. The optimized formulation balances rheological control, mechanical integrity, and cost efficiency, proving effective for leakage mitigation in complex fracture environments.

### 2.2. Evaluation of Physical and Chemical Properties

#### 2.2.1. Microstructure Characterization of Resin Consolidation and Plugging System

**Figure 18:**
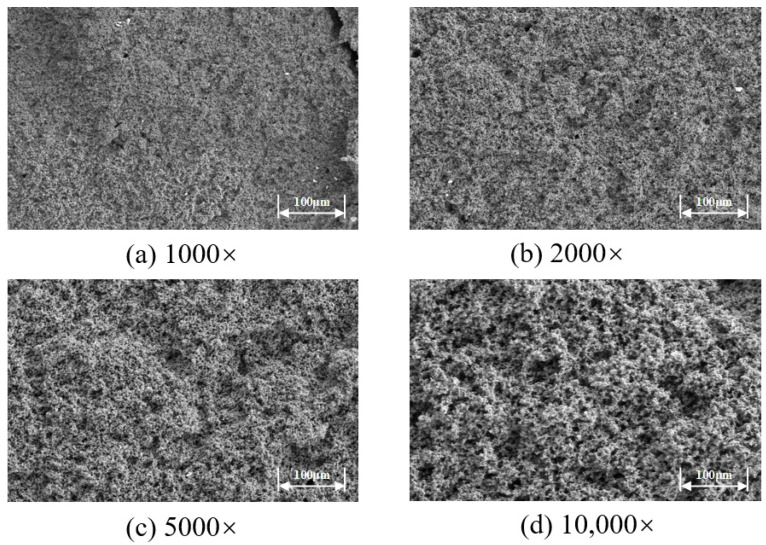
Microstructure of high-temperature and high-strength curable resin.

The microstructure of the high-temperature-resistant, high-strength curable resin was observed using scanning electron microscopy at magnifications of 1000×, 2000×, 5000×, and 10,000×, as shown in Figure 18. The study revealed that the resin formed a chain-like or three-dimensional network structure after curing and crosslinking, with tightly connected resin crystal layers. This microstructure significantly enhanced the material’s toughness and strength. The superior performance originates from the use of low-molecular-weight organosilicon compounds with special structures as resin crosslinkers during the preparation process. These crosslinker molecules contain various active functional groups, such as epoxy, vinyl, amide, and alkoxy groups. One end of these molecules reacts with silanol groups on the surface of inorganic materials (e.g., glass fibers, silicates, metal oxides) to form covalent bonds, while the other end forms covalent bonds with the resin, firmly crosslinking the two originally incompatible materials into a network structure. Additionally, during the curing process, active groups in the resin, such as hydroxymethyl, amide bonds, and dimethylene ether bonds, undergo crosslinking reactions with formaldehyde, further promoting the formation of a three-dimensional spatial network structure. This complex crosslinking mechanism not only imparts excellent high-temperature resistance to the material but also significantly enhances its mechanical strength and toughness.

#### 2.2.2. Infrared Analysis of Resin Consolidation and Plugging System

**Figure 19:**
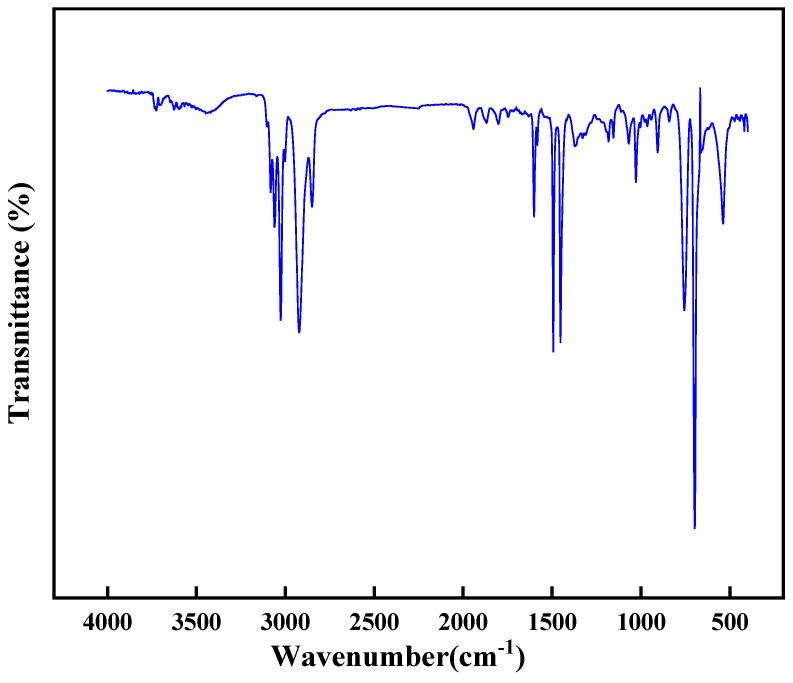
Infrared spectrum analysis of resin gel plugging system.

The chemical structure of the high-temperature-resistant, high-strength curable resin was analyzed using Fourier Transform Infrared Spectroscopy. Before the experiment, the resin was washed with deionized water to remove unreacted components, dried in a vacuum oven, and ground into powder. The sample was prepared using the potassium bromide pellet method. The infrared spectrum was scanned in the range of 4000–400 cm^−1^ at a temperature of 25 °C, with a resolution of 1 cm^−1^ and 8 scans. The experimental results are shown in Figure 19.

The experimental results revealed regular shifts in characteristic peaks of the high-temperature-resistant, high-strength curable resin. According to the Fourier infrared spectra provided, the characteristic peaks of the resin with high-temperature resistance and high strength can be observed in different wavenumber regions. First, peaks near 3000 cm^−1^ are usually associated with stretching vibrations of C-H bonds, indicating that the resin may contain aromatic rings or alkyl chains. Secondly, peaks in the range of 1500–1600 cm^−1^ may be related to the stretching vibration of the C=C bond of the aromatic ring, which is typical of high-temperature-resistant resins because the aromatic structure provides thermal stability. In addition, the peaks in the 1000–1300 cm^−1^ region may be related to the stretching vibration of C-O or C-N bonds, the presence of these functional groups contributes to the cross-linking density, and mechanical strength of the resin. Finally, peaks in the 500–1000 cm^−1^ range may be related to the stretching vibrations of Si-O-Si or Si-C bonds, the presence of which further enhances the high-temperature resistance and chemical stability of the resin. In general, the variation in these characteristic peaks reflects the chemical structure and properties of the resin, and provides important information for the design and optimization of high-performance resin. Specifically, the amide group’s characteristic peak wavenumber progressively decreased, while new structural units including hydroxymethyl (-CH_2_OH) and ether bonds (-O-) emerged. These structural units subsequently diminished as the curing reaction advanced, confirming the resin’s successful synthesis. This behavior aligns with the theoretical mechanism: cross-linking reactions between active groups (hydroxymethyl, amide bonds, and dimethylene ether bonds) and formaldehyde during curing enabled the formation of a robust three-dimensional network structure. This molecular architecture directly correlates with the material’s excellent high-temperature resistance and enhanced mechanical strength, validating its structural design principles.

#### 2.2.3. Thermogravimetric Analysis of Resin Consolidation and Plugging System

**Figure 20:**
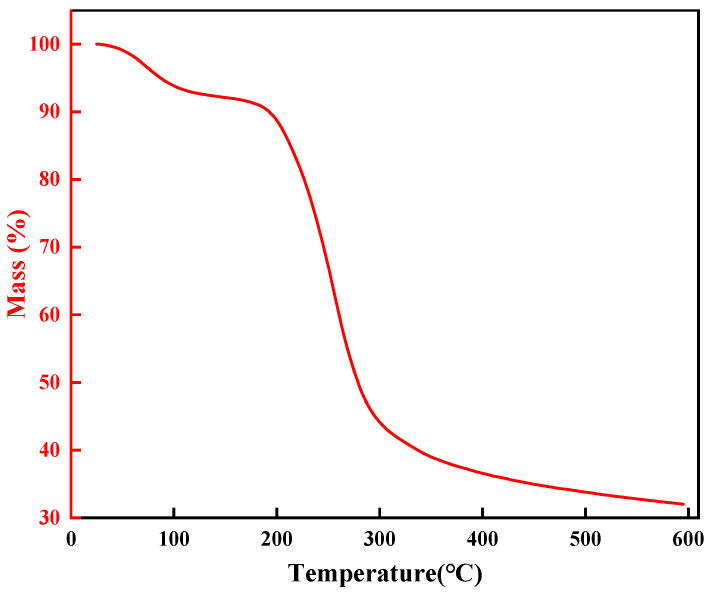
Thermogravimetric analysis of resin gel plugging system.

The thermal stability of high-temperature-resistant, high-strength curable resin powder was analyzed using a thermogravimetric analyzer. Samples were oven-dried at 105 °C to eliminate moisture prior to testing. For each trial, 10–15 mg of dried resin was loaded into a sealed flat-bottom crucible and heated from 25 °C to 600 °C at 20 °C/min under a nitrogen atmosphere (50 mL/min flow rate). Results are presented in Figure 20.

Thermogravimetric analysis (TGA) characterized the thermal degradation of the high-temperature-resistant, high-strength curable resin (Figure 20). The resin exhibited a three-stage weight loss process:

1. Stage 1 (25–175 °C): Initial 8.7% mass loss from evaporation of free/bound water and low-molecular volatiles, with negligible structural impact;
2. Stage 2 (175–355 °C): Major decomposition (52.7% mass loss) occurred as amide groups in the crosslinked network degraded, initiating structural breakdown;
3. Stage 3 (355–465 °C): Final 6.8% mass loss resulted from the fragmentation of hydroxymethyl groups and ether bonds, causing the collapse of the 3D network.

These results clarify the resin’s degradation mechanism and confirm its thermal resilience up to 175 °C, aligning with its designed role in high-temperature applications requiring structural stability.

Based on the comprehensive analysis, the experimental results indicate that the high-temperature-resistant, high-strength curable resin exhibits thermal decomposition stability around 300 °C, demonstrating excellent thermal stability. This property ensures that the resin can maintain its structural and functional integrity even under high-temperature conditions, thereby effectively fulfilling its role in leakage control. If the leakage control agent were to undergo thermal degradation at high temperatures, the breakage of its molecular chains would lead to a decline in performance, rendering it ineffective for sealing purposes. Therefore, the resin’s outstanding thermal stability provides critical assurance for its application in high-temperature environments, ensuring reliable performance in demanding operational conditions.

#### 2.2.4. Laboratory Evaluation of Pressure Plugging Capacity

Using a high-temperature and high-pressure sealing simulation device, the pressure-bearing and sealing performance of the resin gel plugging system was evaluated at 140 °C. In the experiment, the resin was cured at 140 °C for 220 min, with a pumping rate of 10 mL/min and a constant-pressure waiting time of 10 min, followed by a pressure increase. A wedge-shaped fracture with an inlet size of 7 mm and an outlet size of 5 mm was selected to verify the resin’s ability to seal formation fractures under high-temperature and high-pressure conditions.

**Figure 21:**
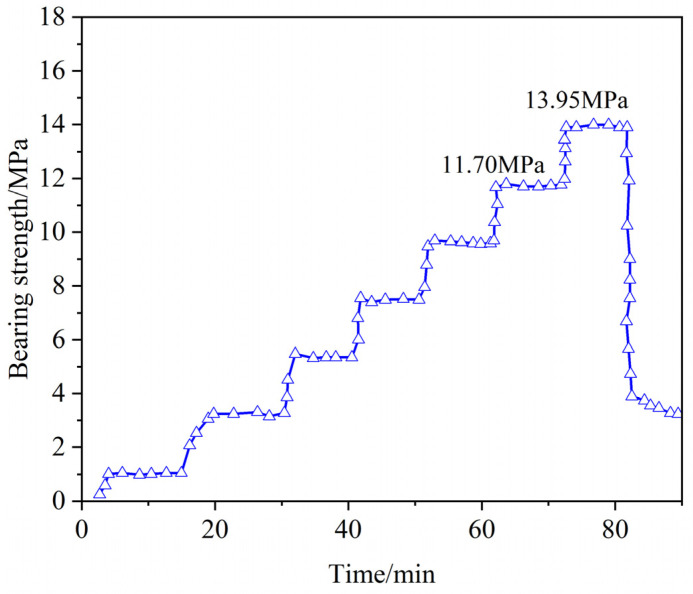
Pressure sealing capacity curve of wedge fracture (inlet 7 mm, outlet 5 mm).

As shown in Figure 21, the resin consolidation leakage control system demonstrated effective initial sealing (no leakage for 10 min at 11.70 MPa). At 13.95 MPa, the sealing layer failed, confirming its high-pressure resistance. The system’s performance derives from the stable crosslinked structure and high-temperature-induced dense packing of the resin. These results underscore its ability to withstand high-temperature, high-pressure conditions, and reliability in complex geological environments, offering critical technical solutions for leakage control in demanding applications.

## 3. Conclusions

(1) Through systematic screening and optimization, this study successfully developed a high-temperature-resistant, high-strength consolidation-type leakage control system. The system is based on urea-formaldehyde resin as the core material (optimal concentration: 25%), combined with a latent curing agent (a mixture of p-toluenesulfonic acid, hexamethylenetetramine, diethanolamine, and ammonium persulfate in a ratio of 1:1:1:2, dosage: 10%), a rheological regulator (0.5% lithium bentonite), and a high-water-loss filler material (Type C, dosage: 10%), forming a synergistic formulation. By precisely controlling the curing time (2–2.5 h) and fluidity, the system addresses the technical challenges of uncontrollable curing and poor pumpability at high temperatures, significantly improving construction safety and leakage control efficiency;
(2) Physicochemical characterization revealed that the cured leakage control system forms a three-dimensional network crosslinked structure. Scanning electron microscopy (SEM) results showed a dense crosslinked structure, endowing the material with excellent mechanical properties (compressive strength: 9.3 MPa). Thermogravimetric analysis (TGA) confirmed that the initial decomposition temperature reaches 241 °C, demonstrating remarkable high-temperature stability. Infrared spectroscopy (FT-IR) revealed the crosslinking reaction mechanism involving hydroxymethyl and ether bonds, verifying the successful preparation of the system. Rheological testing indicated that the system exhibits thixotropic behavior (“shear-thinning, static-thickening”), with a controllable apparent viscosity of 47.5 mPa·s, balancing pumpability and formation retention capability;
(3) Laboratory pressure-bearing and sealing experiments demonstrated that the system, under high-temperature and high-pressure conditions at 140 °C, achieves a pressure-bearing capacity of 13.95 MPa in a wedge-shaped fracture with an inlet width of 7 mm and an outlet width of 5 mm. No leakage was observed after 10 min of waiting, and the sealing layer was dense with outstanding erosion resistance. The high-temperature-resistant, high-strength consolidation-type leakage control system developed in this study exhibits excellent temperature resistance, controllable curing properties, and mechanical strength. It provides a new solution to the challenges of drilling fluid loss in deep and ultra-deep complex fractured formations, demonstrating significant engineering application value and broad prospects for promotion.

## 4. Materials and Methods

### 4.1. Experimental Material

The experimental chemicals used in the study are listed in Table 7.

The experimental instruments used in the study are listed in Table 8.

### 4.2. Preparation Method of Resin Plugging Agent

Using the developed resin material as the core, a high-temperature-resistant, high-strength consolidation-type leakage control system was successfully constructed by introducing key components such as rheological regulators, filler materials, and curing agents. The formulation of the system was precisely optimized with the following component ratios: 25% resin, 10% curing agent, 10% filler material, 0.5% rheological regulator, and 0.05% dispersant. The resin, as the main material, provides excellent adhesion and anti-permeability properties, while the curing agent ensures efficient crosslinking and curing at specific temperatures and times. The filler material enhances the system’s mechanical strength and compressive performance, and the rheological regulator optimizes the system’s fluidity and pumpability during construction. The addition of the dispersant further improves the uniform distribution and stability of the components. Through the synergistic interaction of these components, the system demonstrates exceptional sealing performance and long-term stability under high-temperature and high-pressure conditions, offering reliable technical support for efficient leakage control in complex geological environments.

### 4.3. Rheology

The rheological properties of the resin leakage control system before curing were systematically characterized using a HAAKE MARS 60 (Thermo Fisher Scientific (China) Co., Ltd. (Shanghai, China)) rotational rheometer. A CC41/Ti (Thermo Fisher Scientific (China) Co., Ltd. (Shanghai, China)) rotor (diameter: 41 mm) was selected to ensure the accuracy and reproducibility of the test results. To eliminate the influence of temperature on the measurements, the samples were pre-equilibrated for at least 30 min before testing, and the temperature was strictly controlled within ±0.1 °C using a precision temperature control system. The study focused on the rheological behavior of the resin leakage control system within the linear viscoelastic region, with key measurement parameters including apparent viscosity, storage modulus (G’), and loss modulus (G”) [27]. During the test, the strain range was set from γ = 0.1% to 1000%, and the frequency range varied from 0 to 20 Hz to comprehensively evaluate the system’s rheological properties under different shear conditions. To ensure data reliability, all rheological tests were repeated three times, and the average values were calculated to effectively reduce experimental errors, ensuring scientific rigor and reproducibility of the results.

### 4.4. Infrared Spectrum

The chemical structure of the resin leakage control agent was analyzed using a Fourier Transform Infrared Spectrometer (Nicolet iS50 FT-IR). Before testing, the cured resin leakage control agent sample was thoroughly cleaned with deionized water to remove unreacted components. The sample was then dried in a vacuum oven and ground into a fine powder. The sample was prepared using the potassium bromide (KBr) pellet method. The infrared spectrum was scanned over a range of 4000–400 cm^−1^ at a temperature of 25 °C, with a resolution of 1 cm^−1^, and 8 scans were performed to ensure accurate and reliable results.

### 4.5. Thermogravimetric Analysis

The thermal stability of the chemical bonds in the resin leakage control agent powder was examined using a thermogravimetric analyzer. Prior to testing, the resin leakage control agent was dried in an oven at 105 °C to remove moisture. For each measurement, 10–15 mg of the resin leakage control agent sample was placed in a sealed flat-bottom pan and heated from 25 °C to 600 °C at a rate of 10 °C/min. The experiment was conducted under a nitrogen atmosphere with a flow rate of 50 mL/min.

### 4.6. Scanning Electron Microscope (SEM)

The microstructure of the resin leakage control agent sample was characterized using a scanning electron microscope (SEM). The prepared resin leakage control agent sample was carefully cut to obtain a fresh cross-section, which was then mounted on an aluminum stub and coated with a thin layer of gold to enhance conductivity. SEM imaging was performed at an accelerating voltage of 10 kV.

### 4.7. Compressive Strength

The urea-formaldehyde resin leakage control material was cured and molded into cylindrical specimens with a bottom diameter of 10 mm and a height of 10 mm. The compressive mechanical properties of the resin samples were tested at room temperature using an electronic universal testing machine. The compression speed was set at 3 mm/min, and the stress–strain curve of the resin sample under compression was recorded.

### 4.8. Plugging Performance

The pressure-bearing capacity of the leakage control material is one of the critical parameters for evaluating its sealing effectiveness. A high-temperature and high-pressure fracture physical simulation device was used to study the sealing performance of the curable resin in fractures. The simulated fracture core was made of steel, with a cylindrical shape and a fracture running through the longitudinal section of the steel column. In this experiment, the fracture length was 30 cm, the fracture height was 3 cm, the inlet width was 7 mm, and the outlet width was 5 mm. The leakage control test procedure was as follows:

(a) Adjust the temperature of the heating chamber to the simulated formation temperature of 140 °C;
(b) Place the steel fracture core with the required width into the core holder and apply a confining pressure of 10 MPa;
(c) Inject simulated drilling fluid into the fractured core at a rate of 10.0 mL/min until the core is saturated;
(d) Inject the curable resin solution into the fracture core at a rate of 10.0 mL/min until the resin solution completely exits the fracture outlet and no more water is produced;
(e) Seal the fracture core model and let it stand for 8 h to allow the resin solution to fully react;
(f) Inject simulated drilling fluid into the fractured core in the reverse direction at a rate of 10.0 mL/min. Use data acquisition software to record the injection pressure changes in real time. The maximum pressure achieved represents the pressure-bearing capacity of the resin for the fracture.

## Figures and Tables

**Table 1:** Curing temperatures of different types of resins.

Curing Effect	40 °C	60 °C	80 °C	100 °C
Urea-formaldehyde resin	uncurable	1 h, moderate strength	2 h, the strength is hard	3.46 h, hard strength
Phenolic resin	uncurable	1.5 h, moderate strength	2.5 h, moderate strength	4 h, moderate strength
Epoxy resin	uncurable	0.5 h, the strength is low	1 h, low strength	2.3 h, low strength
Polyvinyl alcohol	Fast curing, low strength	Fast curing, low strength	Fast curing, but almost no strength	A watery, translucent liquid
Melamine resin	uncurable	Milky white, watery, precipitated	Milky white water with precipitate and floating matter	Milky white, watery, partially caked

**Table 2:** Orthogonal optimization test of water-soluble resin preparation.

Number	Urea-Formaldehyde Resin/%	Resorcinol/%	Saccharaldehyde/%	Sodium Dodecyl Sulfate/%	Silicone Crosslinker/%	Settling Time/h
1	The surplus is urea-formaldehyde resin	0.2	0.5	0.3	0.1	36
2		0.2	1.0	0.6	0.3	72
3		0.2	1.5	0.9	0.5	72
4		0.5	0.5	0.3	0.1	132
5		0.5	1.0	0.6	0.3	168
6		0.5	1.5	0.9	0.5	84
7		0.7	0.5	0.3	0.1	48
8		0.7	1.0	0.6	0.3	72
9		0.7	1.5	0.9	0.5	60
10		1.0	0.5	0.3	0.1	36
11		1.0	1.0	0.6	0.3	144
12		1.0	1.5	0.9	0.5	84

**Table 3:** Compressive strength of the resin plugging system formed under high-temperature conditions with different curing agents.

Number	Curing Agent	Curing Temperature/°C	Curing Time/min	Curing Strength/MPa
1	Monoammonium curing agent	130	180	3.93
2	Compound ammonium curing agent	130	180	4.77
3	Latent curing agent	130	180	6.26

**Table 4:** Experimental table of single-factor analysis for optimal dosage of curing agent.

1	400 g Bentonite slurry	150 g Resin powder	4% Curing agent
2	400 g Bentonite slurry	150 g Resin powder	6% Curing agent
3	400 g Bentonite slurry	150 g Resin powder	8% Curing agent
4	400 g Bentonite slurry	150 g Resin powder	10% Curing agent
5	400 g Bentonite slurry	150 g Resin powder	12% Curing agent

**Table 5:** Orthogonal experiment scheme under different additive dosages.

Resin Concentration	Curing Agent Concentration	Curing Temperature/°C	Curing Time/min
10%	10%	130	150
15%	10%	130	150
20%	10%	130	150
25%	10%	130	150
30%	10%	130	150

**Table 6:** Experimental table of single-factor analysis for the optimization of filling materials with high water loss.

Number	Dosage of Resin	Dosage of Amount of Curing Agent	Dosage of Rheological Regulator	Dosage of Filler Material
1	25%	10%	0.5%	1%
2	25%	10%	0.5%	3%
3	25%	10%	0.5%	5%
4	25%	10%	0.5%	7%
5	25%	10%	0.5%	10%
6	25%	10%	0.5%	15%

**Table 7:** Experimental materials.

Reagent Name	Purity	Manufacturer
Urea-formaldehyde resin	99.0%	Shanghai Aladdin Biochemical Technology Co., Ltd. (Shanghai, China)
Phenolic resin	98.0%	Shanghai Maclin Biochemical Technology Co., Ltd. (Shanghai, China)
Epoxy resin	99.0%	Shanghai Sahn Chemical Technology Co., Ltd. (Shanghai, China)
Betaine, one water	99.5%	Shanghai Maclin Biochemical Technology Co., Ltd. (Shanghai, China)
Silane coupling agent KH-570	99.0%	Changzhou Runxiang Chemical Co., Ltd. (Changzhou, China)
Ammonium chloride	99.0%	Shanghai Sahn Chemical Technology Co., Ltd. (Shanghai, China)
Hexamethylenetetramine	98.0%	Shanghai Sahn Chemical Technology Co., Ltd. (Shanghai, China)
Sodium carboxymethyl cellulose	96.0%	Shanghai Sinopharm Group Chemical reagent Co., Ltd. (Shanghai, China)
Barite	99.7%	Shanghai Sinopharm Group Chemical reagent Co., Ltd. (Shanghai, China)
Deionized water		Lab-made

**Table 8:** Experimental instruments.

Instrument Name	Model Number	Manufacturer
Electronic balance	PL602E	Shanghai Mettler Toledo Instruments Co., Ltd. (Shanghai, China)
Infrared spectrometer	Nicolet iS50 FT-IR	Japan Shimadzu (Kawasaki, Japan)
Thermogravimetric analyzer	TGA550	Switzerland Mettler Toledo (Greifensee, Switzerland)
HAAKE rheometer	HAAKE MARS60	Thermo Fisher Scientific (China) Co., Ltd. (Shanghai, China)
HTHP plugging and displacement device	FD-20C	Nantong Xinhuacheng Scientific Research Instrument Co., Ltd. (Nantong, China)
Constant temperature vacuum drying box	DHG-9000	Shanghai Yi Heng Instrument Co., Ltd. (Shanghai, China)

## Data Availability

The original contributions presented in this study are included in the article. Further inquiries can be directed to the corresponding author.
